# MicroRNA-410-3p modulates chondrocyte apoptosis and inflammation by targeting high mobility group box 1 (HMGB1) in an osteoarthritis mouse model

**DOI:** 10.1186/s12891-020-03489-7

**Published:** 2020-07-24

**Authors:** Hong Pan, Huming Dai, Linzhi Wang, Silong Lin, Yuefeng Tao, Yi Zheng, Renyi Jiang, Fan Fang, Yifan Wu

**Affiliations:** Department of Orthopaedics, Affiliated Anqing Hospital of Anhui Medical University, No.352 Ren Min Road, Yingjiang District, Anqing City, 246003 Anhui Province China

**Keywords:** miRNA-410-3p, HMGB1, NF-κB, Osteoarthritis

## Abstract

**Background:**

Osteoarthritis (OA) is the most prevalent type of arthritis, which commonly involves inflammation in the articular cartilage in OA pathogenesis. MicroRNAs (miRNAs) play essential roles in the regulation and pathophysiology of various diseases including OA. MiR-410-3p has been demonstrated to mediate inflammatory pathways, however, the regulatory functions of miR-410-3p in OA remain largely unknown.

**Methods:**

The regulations of miR-410-3p were investigated in OA. Mouse primary chondrocytes and mouse in vivo models were used. The expression levels of miR-410-3p and HMGB1 were measured by qPCR. The transcription activity of NF-κB was assessed by luciferase reporter assay. MTT assay was performed to assess cellular proliferation. Cell apoptosis was evaluated with the Fluorescein Isothiocyanate (FITC) Annexin V assay. Expression levels of proteins were determined by Western blot.

**Results:**

The results demonstrated that miR-410-3p was markedly downregulated in articular cartilage tissues as well as in lipopolysaccharide (LPS)-treated chondrocytes in OA mice. In addition, upregulation of miR-410-3p markedly inhibited LPS-induced apoptosis of chondrocytes. The results also demonstrated that the high mobility group box 1 (HMGB1) was a target of miR-410-3p. LPS-induced upregulated expression of HMGB1 significantly suppressed expression of miR-410-3p. Furthermore, upregulation of miR-410-3p markedly inhibited HMGB1 expression, the nuclear factor (NF)-kB activity and pro-inflammatory cytokines production. Taken together, the results suggested that miR-410-3p targeted HMGB1 and modulated chondrocytes apoptosis and inflammation through the NF-κB signaling pathway.

**Conclusions:**

These findings provide insights into the potential of miR-410-3p/ HMGB1 as therapeutic targets for OA treatment.

## Background

Osteoarthritis (OA) is the most common type of arthritis and there is an estimation of about 240 million people that are affected by OA all over the world [1]. Older adults (> 55-year-old) usually start suffering from OA symptoms and live with this chronic disability because it seriously affects the quality of life [2]. The financial expenditures of OA also pose significant burden to both individual patients and insurers due to health care and social expenditure [3, 4]. Thus, comprehensive elucidation of the molecular mechanisms underlying OA process is of pivotal significance for finding novel therapeutic approaches and treatments.

Various pathologic changes can contribute to OA symptoms, such as the degradation of the articular cartilage and ligaments, inflammation of the synovia, osteophyte formation, hypertrophy of the joint capsule [5]. Dysregulation of chondrocytes, which are the predominant resident cells of the articular cartilage and are responsible for producing and maintaining the cartilaginous matrix, is one of the most common associations with the pathogenesis of OA [6, 7]. Increased cell apoptosis, reduced cell proliferation, cell number and cell viability of chondrocytes, and chondrocytes inflammation are typically involved in the progression of OA [8]. Therefore, fully understanding the regulations of chondrocytes associated with cell apoptosis, inflammation and signaling pathways is a major breakthrough when seeking for more effective therapeutic strategies of OA.

MicroRNAs (miRNAs) are endogenous single strand (21–23 nucleotides) non-coding RNAs that bind to the 3’UTR region of a complementary mRNA sequence, which in turn negatively regulate the targeted gene expression by controlling translation of the targeted RNAs [9]. Increasing numbers of miRNAs have been demonstrated regulating various genes in the human genome [10], and dysregulation of miRNAs are involved in the pathogenesis of various types of diseases including tumorigenesis, neurological and OA [6, 11, 12]. Dysregulation of miR-410-3p has been reported in the progression of breast cancer, pancreatic cancer and prostate cancer [13–15]. For example, miR-410-3p was shown to have protective roles in the pathogenesis of lupus nephritis by suppressing renal fibrosis [16]. However, the fundamental mechanisms of miRNAs underlying OA remain largely unknown.

High mobility group box-1 (HMGB1), which is well identified as a damage-associated molecular pattern (DAMP) protein, is a highly conserved non-histone DNA-binding protein that is expressed in the nucleus of cell eukaryotic cells [17]. HMGB1 has been reported that it can be released into the extracellular space by necrotic cells or cells under stimulation, and transmits the tissue damage signal to adjacent cells [18, 19]. Previous studies have identified that cytoplasmic or extracellular HMGB1 plays roles in the pathogenesis of rheumatoid arthritis including synovium tissue inflammation and joint damage [20, 21]. HMGB1 was also reported to exert pro-inflammatory role contributing to synovitis and articular destruction in OA [22]. HMGB1 can induce the release of tumor necrosis factor-α (TNF-α), interleukin-1β (IL-1β), interleukin 1 (IL-1) and interleukin 6 (IL-6) and it controls the initiation and development of inflammation in various experimental arthritis models [23, 24]. Lipopolysaccharide (LPS) has been considered effective as inducing inflammation simulating the inflammatory environment in OA [25]. LPS is able to bind and activate toll-like receptor-4 (TLR-4), which is one of the TLRs that can identify damage-associated molecular patterns and trigger the inflammatory transcription system, which in turn triggers pro-inflammatory immune response [26].

The goal of this experimental research was to assess the functions of miR-410-3p on the regulation of apoptosis and inflammatory responses of chondrocytes in the progression of OA and the underlying mechanisms. The regulation of HMGB1 and NF-κB pathway by miR-410-3p was also investigated. Findings in the present study would be valuable in elucidating the mechanisms underlying OA.

## Methods

### Experimental animals and creation of OA models

C57BL/6 background male mice (of Jackson origin) aged 8 weeks and weighed 20–25 g were purchased from the Animal Experimental Center of Affiliated Anqing Hospital of Anhui Medical University and were kept at a specific laboratory animal facility in pathogen-free microisolator cages with 12 h/12 h of light/dark at the temperature of 22–24 °C with free access to food and water. Mice were randomly allocated to different groups of treatment. The mice OA model was generated by destabilizing the medial meniscus as previously described [27]. In brief, mice were injected intraperitoneally with 40 mg/kg sodium pentobarbital for anesthesia. Exposure of the hind limbs knee joint was done via the median parapatellar approach and lateral patella dislocation. The medial meniscotibial ligament (MMTL) was transected. Sham-operated mice were used as control. Eight weeks after the surgery, mice were euthanized by cervical dislocation and articular cartilage and synovial fluid were obtained by inserting a needle into the space between bones at the joint. The articular cartilage were subdivision as three parts: one was primary Chondrocytes/articular cartilages of the medial tibial plateau, which was used for qPCR; the second part was primary Chondrocytes/articular cartilages of the medial tibial plateau that was used for western blot; the third part was knee joints of the right hind limbs, articular cartilages of the medial tibial plateau and medial femoral condyle, which was used for histology. The articular cartilages of the medial tibial plateau and synovial fluid were collected and used. All procedures involved in the experiments have been approved by the Animal Protection and Use Committee of Affiliated Anqing Hospital of Anhui Medical University Hospital.

### Cell culture of mice chondrocytes

Mouse primary articular chondrocytes were separated from tissues at knee joint as previously described [28]. Briefly, male mice aged 8 weeks were euthanized by cervical dislocation, and the articular cartilage tissue was carefully removed from the tibial plateau, femoral heads, and femoral condyles, following by rinsing with phosphate buffered saline (PBS). The articular cartilage was then cut off and digested in collagenase D (3 mg/ml; Roche) at room temperature for overnight without rotation. The digestion solution was filtered, and cells were suspended in Dulbecco’s Modified Eagle Medium (DMEM), following with culturing with addition of 10% fetal bovine serum and 1% penicillin/streptomycin with 5% CO_2_ at 37 °C. Cells were used in passage 2–3 and cultured for 2 weeks until 70–80% confluency. All mouse chondrocyte experiments were performed in triplicate.

### Cell transfection

For overexpression, miR-410-3p, miR-505-3p, and miR-129-5p mimics (GenePharma) and negative control vectors were added to the culture medium for transfection of cultured chondrocytes using Lipofectamine™ 2000 (Invitrogen). 24 h later, cells were treated with 100 ng/ml lipopolysaccharide (LPS) (Sigma) for 6 h to induce inflammation.

### qRT-PCR

Total RNAs and miRNAs were isolated using the RNeasy and miRNeasy Mini kits (Qiagen). Total RNAs were reverse transcribed into cDNA with the miScript Reverse Transcription Kit (Qiagen). qRT-PCR was conducted in triplicate using SYBR Green qPCR Assay Kit (Applied Biosystems) for mRNA and TaqMan microRNA Assay Kit (miR-410-3p, #464693; miR-505-3p, #001316; miR-129-5p, #462948; Applied Biosystems) for miRNAs. β-actin was used as an internal control for mRNA. U6 was used as internal control for miRNA. The 2^-ΔΔCt^ method was used for calculating the expression level. Primer sequences were: miR-410-3p, F, 5′-GGUACCUGAGAAGAGGUUGU-3′; miR-410-3p, R, 5′-GAUGGCCUGUUUUCAGUACC-3′; miR-505-3p, F, 5′-GCGAGCACCGTCAACACT-3′; miR-505-3p, R, 5′-TGGTGTCGTGGAGTCGGC-3′; miR-129-5p, F, 5′-GCGGCTTTTTGCGGTCTGG-3′; miR-129-5p, R, 5′-GTGCAGGGTCCGAGGT-3′; U6, F, 5′-CTCGCTTCGGCAGCACA-3′; U6, R, 5′-AACGCTTCAGAATTTGCGT-3′; HMGB1, F, 5′-GATGGGCAAAGGAGATCCTA-3′; HMGB1, R, 5′-CTTGGTCTCCCTTTGGGG-3′; β-actin, F, 5′-CCCATCTATGAGGGTTACGC-3′; β-actin, R, 5′-TTTAATGTCACGCACGATTTC-3′.

### Cell proliferation assessment

MTT assay was performed to assess cellular proliferation. Cells were seeded into 96-well plate at a density of 1 × 10^5^ (cells/well) for incubation of 24 h. 100 μg of MTT (Sigma) was added for incubation at 37 °C in dark for 4 h. The MTT-containing medium was discarded and 100 μl of DMSO (Sigma) was added for dissolving formazan product. The solution was shaked in the dark at room temperature for 15 min and absorbance value was determined with 490 nm wavelength on the Bio-Tek Microplate Reader instrument.

### Apoptosis detection assay

Cell apoptosis was evaluated with the Fluorescein Isothiocyanate (FITC) Annexin V Apoptosis Detection Kit (Becton-Dicknson Biosciences) following the manufacturer’s protocol. In brief, cells were thoroughly washed with PBS after treatment, trypsinized, and subsequently suspended in mixture buffer. 195 μl of cell suspension was incubated with 5 μl of FITC-conjugated Annexin-V binding buffer in the dark. Supernatant was removed after 15 min of incubation, cells were washed with mixture buffer and stained in mixture buffer containing 20 μg/ml PI solution in the dark for 30 min. A FACScan flow cytometer (FACSCalibur) was used to determine cell apoptosis.

### Elisa

Protein levels in culturing supernatant and synovial fluid were determined by the Enzyme-Linked Immunosorbent Assay **(**ELISA) kit (R&D Systems) following the manufacturer’s protocol.

### Dual-luciferase reporter assay

A fragment of the 3′ UTR of HMGB1 that contains the predicted miR-410-3p binding site was amplified and cloned into the dual-luciferase reporter vector pmirGLO (Genechem). The HMGB1 3′-UTR mutant (mutation was generated in the HMGB1 3′-UTR by mutating seed matching sequence) luciferase reporter constructs were also constructed. A negative control luciferase vector (NC) was also constructed. The three vectors were co-transfected with miR-410-3p mimics or negative control mimic into 293 T cells (ATCC) using Lipofectamine™ 2000 reagent (Invitrogen). Cells were harvested after 48 h of transfection. Luciferase activities were detected using the Dual-Luciferase Reporter Assay System (Promega).

### NF-κB luciferase reporter assay

The transcription activity of NF-κB was assessed using an NF-κB promoter luciferase vector (Promega). Briefly, cells were cotransfected with NF-κB promoter luciferase vector and miR-410-3p mimics or miR-negative control mimic. After 48 h of culturing, cells were treated with LPS cultured for another 6 h. Luciferase activities were determined with the Dual-Luciferase Reporter Assay System (Promega).

### Western blot

Total proteins of cells and mouse articular cartilage tissues were isolated with RIPA buffer containing 1% protease inhibitors (Sigma-Aldrich). Equal amount of each protein sample was loaded on 12.5% SDS-PAGE and then transferred to PVDF membranes (Millipore). Blocking was done by incubating the membranes with TBST containing 5% slim milk at 37 °C for 2 h and the corresponding primary antibodies at 4 °C for overnight. Membranes were then incubated with HRP-conjugated secondary antibodies (1:2000, Abcam) at RT for 1 h. Proteins were detected with an enhanced chemiluminescence (ECL) kit (Pierce). Primary antibodies used were anti-β-actin (1:1000, Abcam, ab8227), anti-p65 (1:1000, Abcam, ab16502), anti-IkBα (1:1000, Abcam, ab76429), and anti-HMGB1 (1:1000, Abcam, ab77302). Proteins were quantitatively analyzed and normalized relative to β-actin using Image-Pro Plus software (v6.0) (Media Cybernetics).

### In vivo experiments

A total of 18 mice were randomly divided into 3 groups, and each experimental group included 6 mice (*n* = 6), including the OA mice from infection with miR-410-3p-expressing lentivirus (OA + LV-miR-410-3p mimics), OA mice from infection with NC lentivirus (OA + LV-NC) (Research-Bio-Tech Co., Ltd., Shanghai, China), and the Sham-operated group. The OA model was induced by destabilizing the medial meniscus surgery. Mice were killed 8 weeks after the surgery and were infected with LV-miR-410-3p mimics or LV-NC by intra-articular injection (1 × 10^9^ plaque-forming units) of a total volume of 10 μL, which was performed with an interval of 2 days, 2 weeks after OA surgery, and then were sacrificed at 6 weeks later. The articular cartilages and the synovial fluid were stored for further experiments.

### Histological assessment

All 3 groups’ articular cartilage tissues specimens were fixed in 4% paraformaldehyde for 24 h at room temperature. The specimens were decalcified in neutral 10% EDTA solution, gradient dehydrated, and embedded in paraffin wax, and sectioned (5 μm). And the sections were stained with Safranin O-Fast Green, after that, the degree of articular cartilage lesions was scored by three independent observers according to the Glasson scoring principle [29]. The Osteoarthritis Research Society International (OARSI) score of cartilage OA range was 0–6, and the higher the score, the more severe the joint injury.

### Statistical analysis

Statistical analysis was performed using the software Statistical Program for Social Sciences version 19.0 (SPSS Inc., USA). Data values were mean ± standard deviation (SD) of three independent experiments. The independent-samples T-test was performed for the significance of difference between two groups. One-way ANOVA analyses and Bonferroni’s post-hoc test were performed for the significance of difference among multiple-group comparisons. Statistically significant difference was considered when *P <* 0.05.

## Results

### Identification and verification of candidate HMGB1-associated miRNAs

**Fig. 1:**
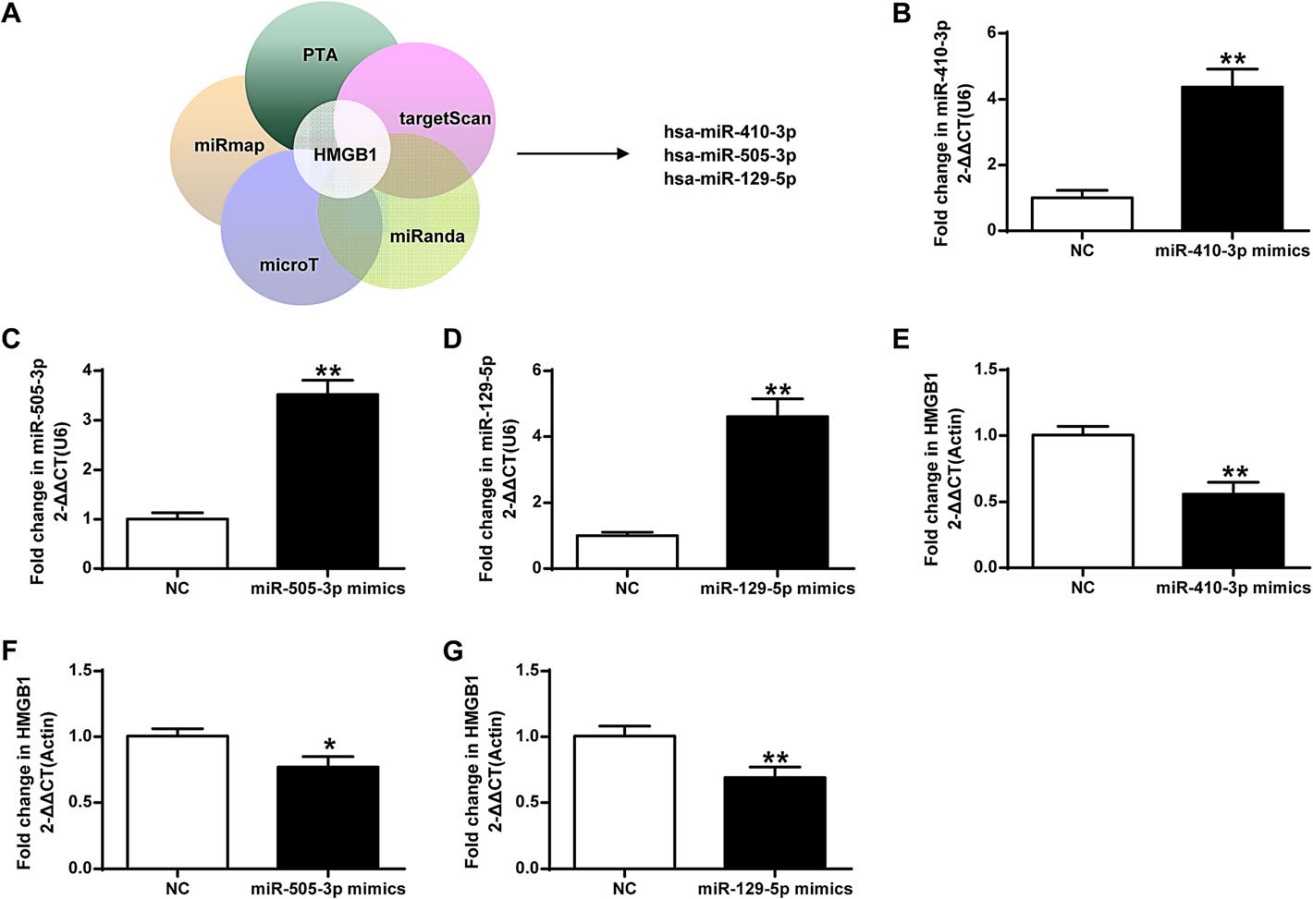
Identification and verification of candidate HMGB1-associated miRNAs. **a**, miRNAs that were identified as candidates associated with HMGB1 based on ‘online available bio-informatic tools including PTA, miRmap, microT, miRanda and targetScan. **b**, qRT-PCR detection of transcript levels after overexpression of the candidate miRNAs miR-410-3p, (**c**), miR-505-3p and (**d**), miR-129-5p in mouse primary chondrocytes. E, qRT-PCR detection of transcript levels of HMGB1 with the overexpression of miR-410-3p, (**f**), miR-505-3p and (**g**), miR-129-5p. The value of the control was set at ‘1’ in qRT-PCR analysis. *P < 0.05, **P < 0.01 vs NC

Three miRNAs, miR-410-3p, miR-505-3p and miR-129-5p, were considered as candidate miRNAs that were associated with HMGB1 based on ‘online available bio-informatic tools, including PTA, miRmap, microT, miRanda and targetScan (Fig. 1a). The miRNAs mimics of three corresponding candidate miRNAs were transfected into mouse primary chondrocytes for miRNA overexpression. Compared to the negative control (NC) group, the expression of miR-410-3p, miR-505-3p and miR-129-5p was significantly upregulated (*P <* 0.01) (Fig. 1b-d). On the contrary, compared to the NC group, HMGB1 mRNA expression was significantly down-regulated with the overexpression of miR-410-3p, miR-505-3p and miR-129-5p (*P <* 0.05; *P <* 0.01), with the most down-regulation by miR-410-3p (*P <* 0.01) (Fig. 1e-g), which was selected for further experiments. The results suggested that HMGB1 might be negatively regulated by miR-410-3p in chondrocytes.

### Overexpression of miR-410-3p protects against LPS-induced injury in mouse chondrocytes

**Fig. 2:**
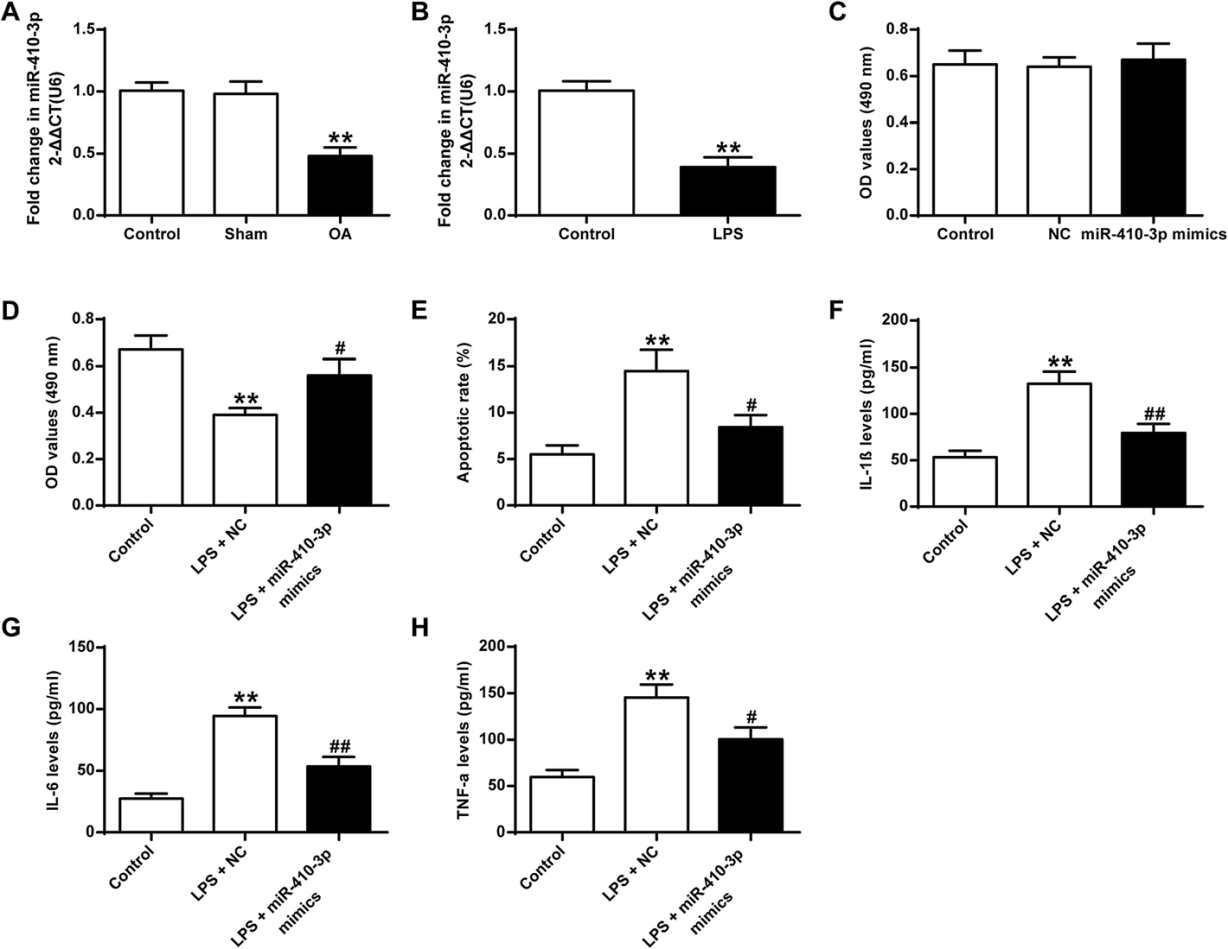
Differential expression of miR-410-3p in OA mice and the effect of overexpressing miR-410-3p in LPS-induced injury in mouse primary chondrocytes. **a**, qRT-PCR detection of transcript levels of miR-410-3p in articular cartilage tissues in OA mice, Sham-operated mice and control mice. **b**, qRT-PCR detection of transcript levels of miR-410-3p in LPS-treated and control chondrocytes. **c**, MTT assay detecting chondrocyte proliferation without LPS treatment. **d**, MTT assay detecting chondrocyte proliferation after LPS treatment and overexpression of miR-410-3p. **e**, Annexin V-PI staining assay detecting cell apoptosis in control, LPS treated chondrocyte (LPS + NC), and overexpression of miR-410-3p in LPS treated chondrocyte (LPS + miR-410-3p mimics). **f**, ELISA detecting the production of IL-1β, (**g**), IL-6 and (**h**), TNF-α in control, LPS + NC and LPS + miR-410-3p mimics on the supernatant of mouse primary chondrocytes. Each experimental group included 6 mice (*n* = 6). The value of the control was set at ‘1’ in qRT-PCR analysis. **P < 0.01 vs Control or Sham-operated; #*P* < 0.05, ##*P* < 0.01 vs LPS + NC

The potential function of miR-410-3p was investigated by detecting its expression level in articular cartilage in an OA mouse model. As shown in Fig. 2, expression of miR-410-3p was remarkably down-regulated in articular cartilage from OA mice, in comparison with that in the control and Sham-operated mice (*P <* 0.01). Also, expression of miR-410-3p was also markedly down-regulated in LPS-treated chondrocytes (*P <* 0.01) (Fig. 2b). Results also showed that without LPS treatment, overexpression of miR-410-3p had no marked effect on chondrocyte proliferation (*P* > 0.05) (Fig. 2c). However, chondrocyte proliferation was markedly reduced after LPS treatment (*P <* 0.01), and upregulation of miR-410-3p remarkably rescued the adverse effect of LPS treatment (*P <* 0.05) (Fig. 2d). Furthermore, overexpression of miR-410-3p also significantly inhibits LPS-induced apoptosis (*P <* 0.05) (Fig. 2e). Overexpression of miR-410-3p significantly decreased the production of pro-inflammatory cytokines including IL-1β, IL-6, and TNF-α that were significantly induced by LPS treatment (*P <* 0.05; *P <* 0.01) (Fig. 2f-h). The results together indicated that miR-410-3p has protective effects against LPS-induced cell apoptosis as well as pro-inflammatory cytokine production in chondrocytes.

### MiR-410-3p directly targets HMGB1

**Fig. 3:**
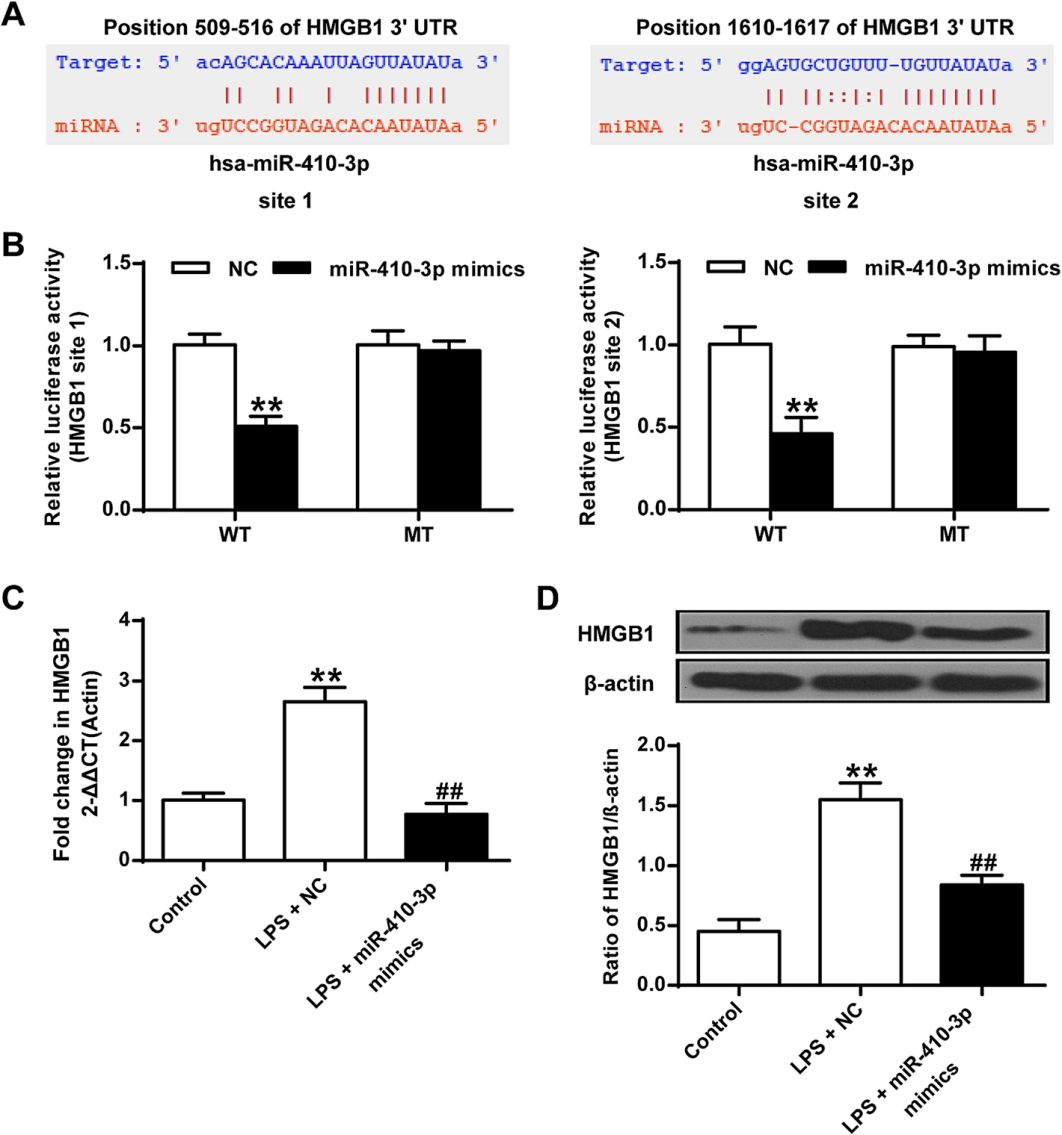
MiR-410-3p targets HMGB1 in mouse primary chondrocytes. **a**, Two predicted binding sites of miR-410-3p in the 3′ UTR region of HMGB1. **b**, Relative dual-luciferase activity in mouse primary chondrocytes by cloning a fragment of the wild type (WT) or mutant (MT) 3′ UTR of HMGB1 for both binding sites into the luciferase reporter vector and followed by cotransfection with miR-410-3p mimics or NC mimics into 293 T cells. **c**, qRT-PCR detection of transcript levels of HMGB1 in control, LPS treated mouse primary chondrocyte (LPS + NC) and overexpression of miR-410-3p in LPS treated mouse primary chondrocyte (LPS + miR-410-3p mimics). **d**, Western blot analysis detecting protein expression of HMGB1 in control, LPS + NC and LPS + miR-410-3p mimics mouse primary chondrocytes. β-actin was used as a loading control. The value of the control was set at ‘1’ in qRT-PCR analysis. **P < 0.01 vs NC or Control; ##*P* < 0.01 vs LPS + NC

Bioinformatics analysis was performed using online tools including PTA, miRmap, microT, miRanda and targetScan, and two putative binding sites were identified for miR-410-3p in the 3′ UTR region of HMGB1 (Fig. 3a). To verify this prediction, luciferase reporter assay were conducted by cloning a fragment of the wild type (WT) or mutant 3′ UTR of HMGB1 in the two predicted binding sites into the luciferase gene vector, followed by cotransfection with miR-410-3p mimics or NC mimics into 293 T cells. As shown in Fig. 3b, overexpression of miR-410-3p significantly decreased luciferase activities of the wild type but not the mutant of HMGB1 3′ UTR vector (*P* < 0.01) for both sites. In addition, upregulation of miR-410-3p significantly suppressed expression level of HMGB1 (*P* < 0.01) (Fig. 3c) and protein level (*P* < 0.05) (Fig. 3d) in LPS treated chondrocytes. These results indicated that miR-410-3p directly targets HMGB1.

### MiR-410-3p inhibited NF-κB pathway and LPS-induced injury in mouse chondrocytes

**Fig. 4:**
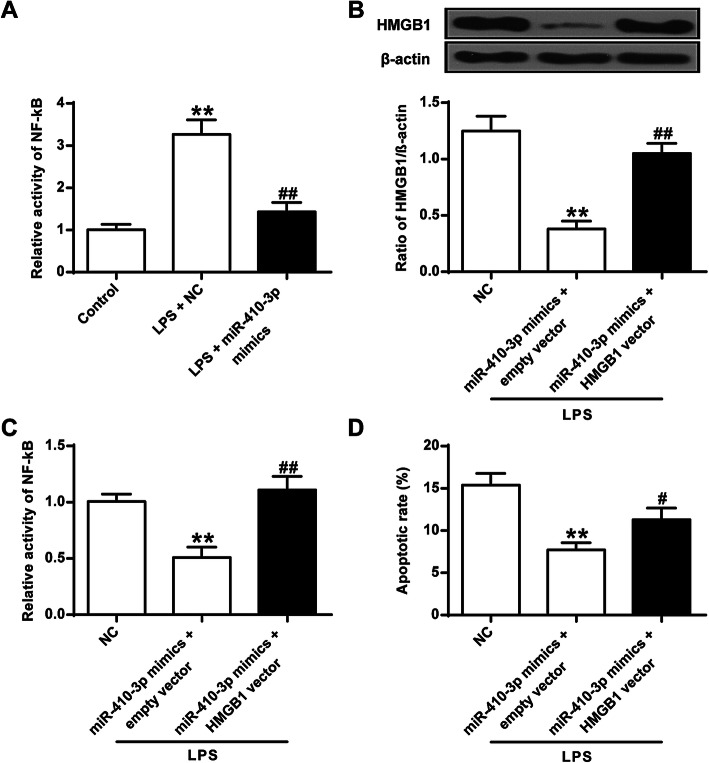
MiR-410-3p regulates the NF-κB pathway via HMGB1. **a**, Effect of overexpression of miR-410-3p on LPS-induced NF-κB activity in mouse primary chondrocytes. **b**, Western blot analysis detecting protein expression of HMGB1 in control, miR-410-3p mimics + empty vector, and miR-410-3p mimics + HMGB1 vector mouse primary chondrocytes. **c**, Effect of miR-410-3p on NF-κB activity after restoring HMGB1 expression. **d**, Annexin V-PI staining assay detecting cell apoptosis after restoring HMGB1 expression. **P < 0.01 vs Control or NC; #*P* < 0.05, ##*P* < 0.01 vs LPS + NC or miR-410-3p mimics + vector

To further explore underlying mechanisms of the protective role of miR-410-3p in chondrocytes, the NF-κB pathway was investigated. The results demonstrated that the LPS-induced NF-κB activity was significantly inhibited with the overexpression of miR-410-3p (*P* < 0.01) (Fig. 4a). To assess if miR-410-3p regulates the NF-κB pathway via HMGB1, the expression of HMGB1 was significantly restored after rescue (*P* < 0.01) (Fig. 4b). And the restoration of HMGB1expression markedly attenuated the suppression of miR-410-3p on NF-κB activity (*P* < 0.01) (Fig. 4c). In addition, restored HMGB1 expression also abolished the protective effect of miR-410-3p on LPS-induced cell apoptosis (*P* < 0.05) (Fig. 4d). The results suggested that miR-140-3p protects chondrocytes from LPS-induced injury by inhibition of the NF-κB pathway through HMGB1.

### MiR-410-3p suppresses pro-inflammatory cytokine production and NF-κB pathway in vivo

**Fig. 5:**
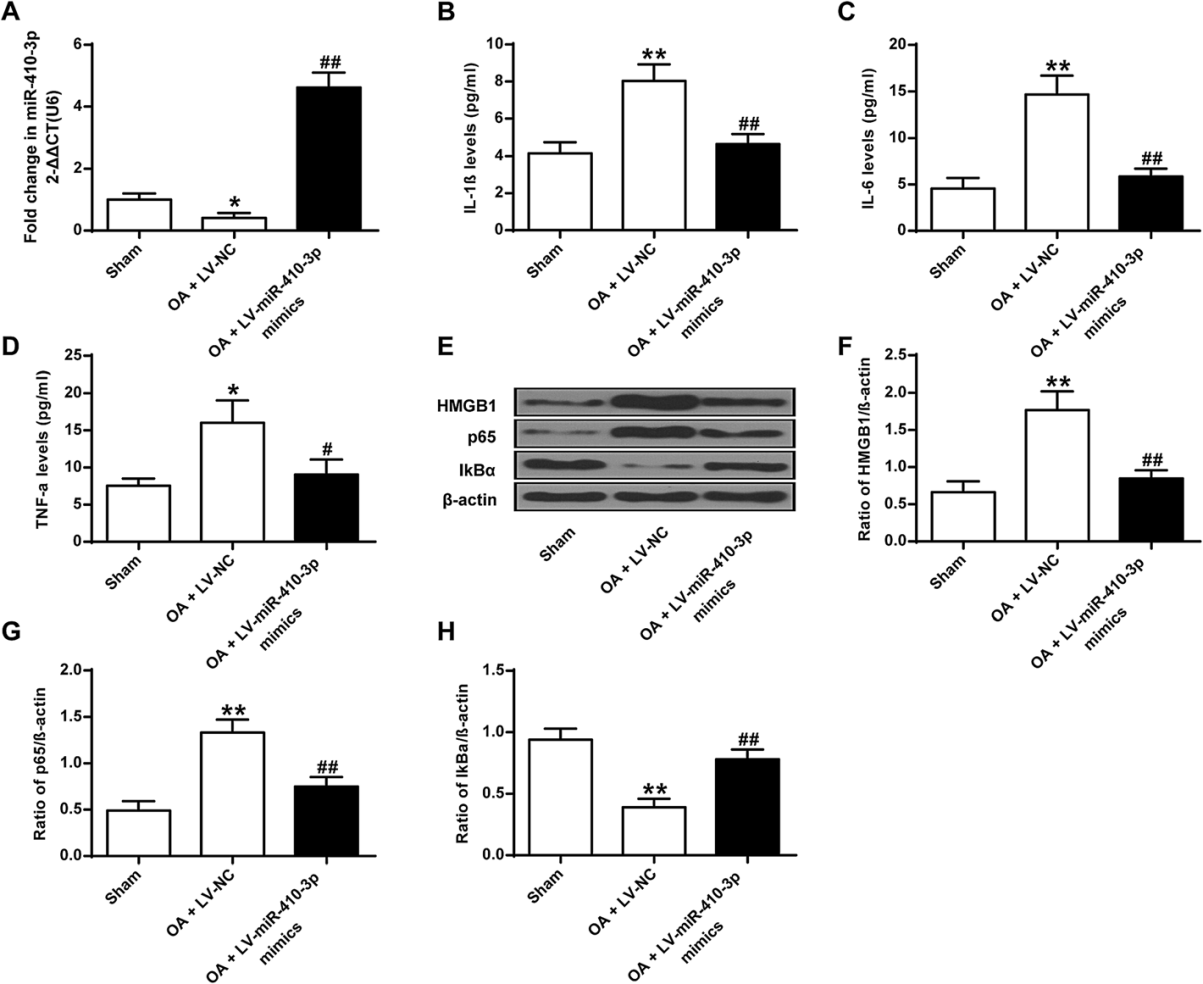
Effect of miR-410-3p on proinflammatory cytokines and the NF-κB pathway in vivo. **a**, qRT-PCR detection of transcript levels of miR-410-3p in articular cartilage samples from Sham-operated mice, OA mice infected with control lentivirus (OA + LV-NC), and OA mice infected with miR-410-3p-expressing lentivirus (OA + LV-miR-410-3p mimics). **b**, ELISA detecting the production of IL-1β, (**c**), IL-6 and (**d**), TNF-α on synovial fluid in Sham-operated, OA + LV-NC and OA + LV-miR-410-3p mimics mice. **e**, Western blot analysis showing representative blotting band intensities of detecting protein expression of HMGB1, IkBα, and p65 in chondrocytes of Sham-operated, OA + LV-NC and OA + LV-miR-410-3p mimics mice. **f**, Quantification data of western blot analysis showing the expression of HMGB1, (**g**), IkBα, and (**h**), p65 in Sham-operated, OA + LV-NC and OA + LV-miR-410-3p mimics mice. β-actin was used as a loading control. The value of the control was set at ‘1’ in qRT-PCR analysis. Each experimental group included 6 mice (*n =* 6). *P < 0.05, **P < 0.01 vs Sham-operated; #*P* < 0.05, ##*P* < 0.01 vs OA + LV-NC

The protective roles of miR-410-3p in vivo was further validated, miR-410-3p-expressing lentivirus was used to infect OA mice (*n* = 6). The results demonstrated that in comparison with Sham-operated mice, miR-410-3p expression was markedly decreased in OA + LV-NC mice (*P* < 0.05), but significantly increased in OA + LV-miR-410-3p mimics mice (*P* < 0.01) (Fig. 5a). Upregulated expression of miR-410-3p in vivo markedly decreased production of IL-1β, IL-6, and TNF-α (*P* < 0.01; *P* < 0.05) that were markedly induced in OA mice (*P* < 0.01) (Fig. 5b-d). In addition, the highly upregulated expression levels of HMGB1 and p65 in OA mice were remarkably inhibited by overexpression of miR-410-3p (*P* < 0.01), while the highly suppressed expression of IkBα, which is important for NF-κB signal transduction [30], was significantly increased by LV-miR-410-3p (*P* < 0.01) (Fig. 5e-h). These data indicated that miR-410-3p inhibited NF-κB pathway and alleviated OA through HMGB1.

### Overexpression of miRNA-410-3p significantly decreased cartilage damage of OA in vivo

**Fig. 6:**
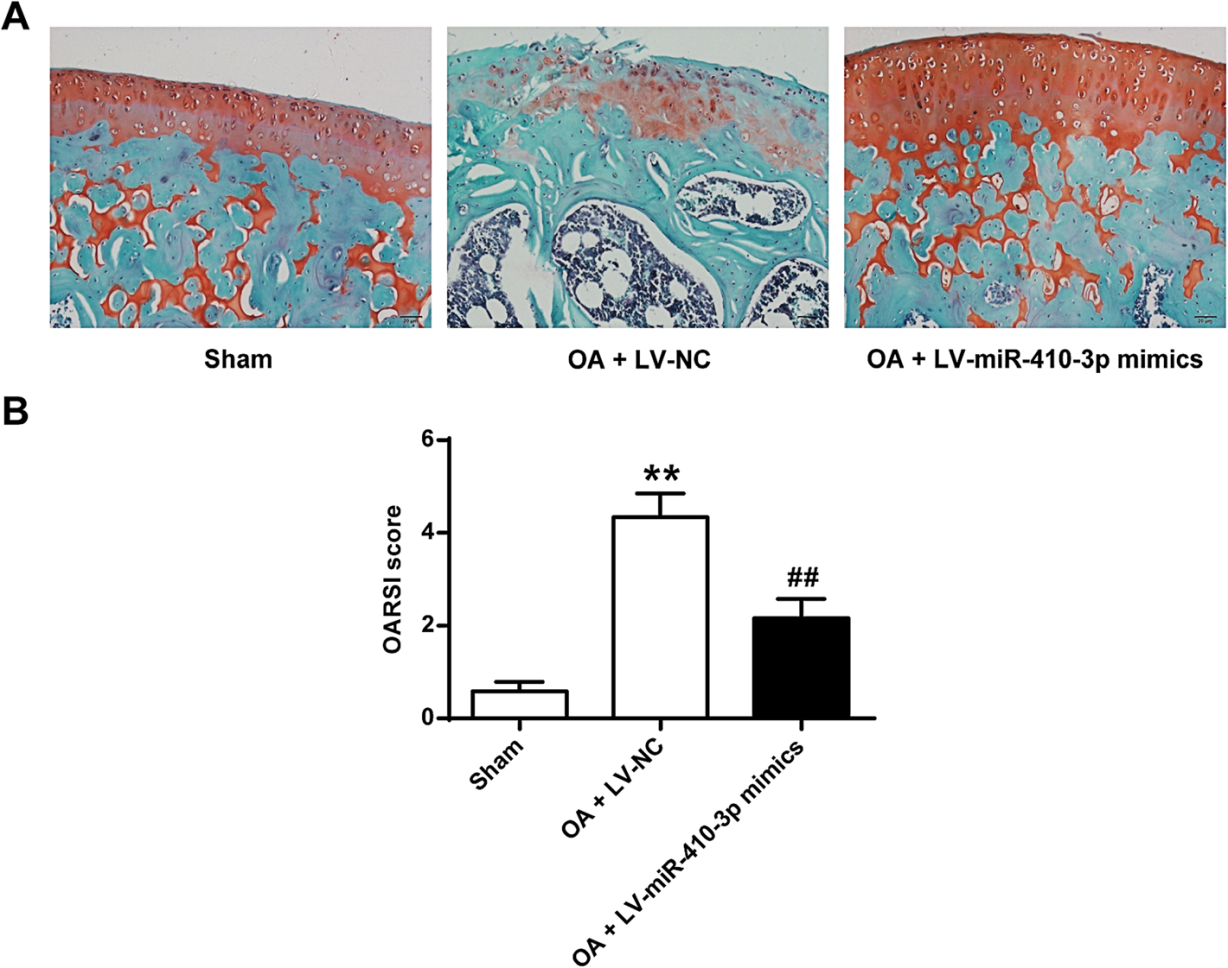
Overexpression of miRNA-410-3p significantly decreased cartilage damage of OA in vivo. **a**, Histological safranin O-Fast Green staining of knee joint sections in Sham-operated, OA + LV-NC and OA + LV-miR-410-3p mimics mice. **b**, Quantitative OARSI scores of the histological staining. We used highest score from the medial femoral condyle (MFC) and the medial tibial plateau (MTP). Scores were ranging from 0 to 6 based on cartilage structural damage. Each experimental group included 6 mice (*n* = 6). The statistical analysis was done by One-way ANOVA analyses with Bonferroni’s post-hoc test, *P*-values, vs sham (F [2, 15]=134.298, *P* = 0.000; vs OA + LV-NC, F [2, 15]=134.298, *P =* 0.000)

To further investigate the effect of lentiviral overexpression of miRNA-410-3p in the articular cartilage of mice, in vivo OA mouse experiment was performed. The results showed that OA mice infected with LV-miR-410-3p mimics showed less damage on superficial layer with decreased cartilage surface erosion and increased safranin-O staining in the superficial layer (Fig. 6a), as compared with OA mice infected with LV-NC. The quantitative data also showed that overexpression of miR-410-3p significantly decreased the cartilage damage in an OA mouse model in vivo, which could be read in the histologic scores of OA (Fig. 6b), we used the highest score from the medial femoral condyle (MFC) and the medial tibial plateau (MTP). These results demonstrated that overexpression of miRNA-410-3p significantly decreased cartilage damage of OA in vivo.

## Discussions

Although enormous efforts have been made to study OA, the molecular mechanisms underlying OA progression are largely unknown, and currently there are not many novel interventions to effectively decelerate its progression. There is an estimation that the incidence of OA will be increasing and affecting around 25% of the adult population in the US by 2020 [31]. Due to the multi-factorial etiology of OA and its high risk of causing morbidity and severe physical limitations on the patients, seeking for more effective therapeutic strategies for OA treatment is of critical importance.

Accumulating evidence has shown that miRNAs as regulators of other signaling pathways are essentially involved in the regulation of extracellular matrix remodeling, mesenchymal stromal cell differentiation and pro-inflammatory mediator releases, which are key factors involved in the progression of osteoarthritis [32]. It has been reported that abnormal expression of miR-410-3p is present in different kinds of diseases and regulates many important biological processes in inflammation, angiogenesis and tumorigenesis, including cell proliferation, invasion and migration, apoptosis and stem cell differentiation [16, 33, 34]. MiR-410-3p was shown to have protective functions by suppressing cell proliferation and invasion in breast cancer and pancreatic cancer [15, 35]. However, there are also studies showing that miR-410-3p enhances cancer cell growth in lung cancer and colorectal cancer [36, 37]. One study reported that miR-410-3p regulates the differentiation of bone marrow mesenchymal stem cells in the development of osteoarthritis [38]. MiR-410-3p was also reported to regulate synoviocyte proliferation and apoptosis in rheumatoid arthritis [39]. In this study, the protective function of miR-410-3p was identified in OA. MiR-410-3p was down-regulated in LPS-treated chondrocytes and the articular cartilage tissues in OA mice, and overexpression of miR-410-3p could protect chondrocytes and OA mice from inflammation and injury induced by LPS.

MiR-410-3p was found to protect chondrocytes by regulating inflammatory signaling via targeting of HMGB1 in our research. Overexpression of miR-410-3p remarkably inhibited the upregulated expression of HMGB1 induced by LPS. The protective effect of miR-410-3p was largely receded after rescuing HMGB1 expression. Previous study has shown that upregulation of miR-410-3p enhanced the chemoresistanc of pancreatic ductal adenocarcinoma by targeting HMGB1 and suppressing HMGB1-mediated autophagy [40]. Wang et al. identified that an inflammation-related miRNA, miR-142-3p, inhibits inflammation and the OA progression by targeting HMGB1 in OA mice in vitro and in vivo [41]. A recent study reported that miR-140-5p has a relatively similar role to miRNA-410-3p, which is that it affects chondrocyte proliferation, apoptosis and inflammation by targeting HMGB1 in osteoarthritis [42]. HMGB1, with its nuclear function in lots of DNA activity-associated processes such as DNA replication, transcription and repair, is an essential molecular target in various human diseases [43]. In addition, it can also activate the pro-inflammatory signaling when releasing from the nucleus to cytoplasm or extracellular matrix [44]. Recently, an increasing number of evidence has revealed the pathogenic function of HMGB1 in OA. Expression of HMGB1 was upregulated in osteoarthritic cartilage chondrocytes as well as in synovial fluid in OA patients [45, 46]. The increased expression of HMGB1 can enhance the inflammatory phenotypes in OA [47], and the suppression of HMGB1 expression has shown anti-inflammatory effects [48]. However, in addition to the expression of miR-410-3p in chondrocytes, our findings will be more comprehensive with additional experiments such as in situ hybridization (ISH) or immunohistochemistry (IHC) to localize the expression of miR-410-3p in further studies, which would expand the understandings of regulation between miR-410-3p and HMGB1.

The NF-κB family of transcription factors contains p50, p52, p65, RelB, v-Rel, and c-Rel, and the most common heterodimers of NF-κB are consisted of p50 (RelA) and p65 (NF-κB1) subunits [49, 50]. The NF-κB signaling was extensively investigated due to its pathogenic role in various diseases. NF-κB has been demonstrated to regulate cell proliferation, apoptosis, metastasis and tumorigenesis [51]. NF-κB is the most representative transcription factor that is activated by cellular stimulation with LPS, which leads to nuclear translocation of NF-κB and in turn to activate downstream genes such as TNF-α, IL-1β and IL-6 [52]. The binding of HMGB1 and its cell surface receptors is able to activate the NF-κB pathway, resulting in the activation of TNF-α, IL-1β and IL-6 [53]. The mediation between HMGB1 and the NF-κB pathway has been shown to have important regulatory functions in the processes of inflammation in different diseases [54, 55]. The results in this study demonstrated that the suppression of HMGB1 by miR-410-3p markedly suppressed the NF-κB signaling pathway in chondrocytes. Furthermore, the highly upregulated expression levels of HMGB1 as well as the transcription targets of NF-κB including IL-1β, IL-6, and TNF-α were remarkably inhibited by overexpression of miR-410-3p. The findings indicated that miR-410-3p/HMGB1 potentially functions anti-inflammatory in OA progression possibly via the NF-κB signaling pathway. In future studies, it would be more interesting to include a control condition in which an inhibitor of miRNA-410-3p is used, to confirm the effects of miR-410-3p in OA. In conclusion, our study demonstrated that miR-410-3p was an effective inhibiting agent of HMGB1. And miR-410-3p directly binds to the 3′-UTR of HMGB1 to inhibit its expression. Overexpression of miR-410-3p protected chondrocytes from LPS-induced injury. The protective function of miR-410-3p in interfering OA progression was also demonstrated in vivo in OA mice. Our study provides valuable insights into the application potential of regulating miR-410-3p and HMGB1 as therapeutic targets for OA treatment.

## Supplementary information

**Additional file 1 Western blot of** Fig. 3d**: HMGB1 in control, LPS + NC and LPS + miR-410-3p mimics mouse primary chondrocytes, β-actin was used as a loading control. Western blot of** Fig. 4b**: HMGB1 in control, miR-410-3p mimics + empty vector, and miR-410-3p mimics + HMGB1 vector mouse primary chondrocytes. Western blot of** Fig. 5e**: HMGB1, IkBα, and p65 in chondrocytes of Sham-operated, OA + LV-NC and OA + LV-miR-410-3p mimics mice.**

## Data Availability

The analyzed data sets generated during the study are available from the corresponding author on reasonable request.

## Abbreviations

OA: Osteoarthritis
miRNAs: MicroRNAs
LPS: Lipopolysaccharide
HMGB1: High mobility group box 1
NF: Nuclear factor
DAMP: Damage associated molecular pattern
TNF-α: Tumor necrosis factor-α
IL-1β: Interleukin-1β
IL-1: Interleukin 1
IL-6: Interleukin 6
TLR-4: Toll-like receptor-4
MMTL: Medial meniscotibial ligament
FITC: Fluorescein Isothiocyanate
ECL: Enhanced chemiluminescence
LV: Lentivirus
SD: Standard deviation
