# Supplementary material for: MicroRNA-410-3p modulates chondrocyte apoptosis and inflammation by targeting high mobility group box 1 (HMGB1) in an osteoarthritis mouse model
Source: BMC Musculoskelet Disord. 2020 Jul 24;21:486. doi: 10.1186/s12891-020-03489-7 (PMC7379779; doi:10.1186/s12891-020-03489-7)
Supplement: Supplementary file 1 — Additional file 1 Western blot of Fig. 3d: HMGB1 in control, LPS + NC and LPS + miR-410-3p mimics mouse primary chondrocytes, β-actin was used as a loading control. Western blot of Fig. 4b: HMGB1 in control, miR-410-3p mimics + empty vector, and miR-410-3p mimics + HMGB1 vector mouse primary chondrocytes. Western blot of Fig. 5e: HMGB1, IkBα, and p65 in chondrocytes of Sham-operated, OA + LV-NC and OA + LV-miR-410-3p mimics mice. [file 12891_2020_3489_MOESM1_ESM.docx]

**Western blot of Figure 3D: HMGB1 in control, LPS + NC and LPS + miR-410-3p mimics mouse primary chondrocytes, β-actin was used as a loading control**

**
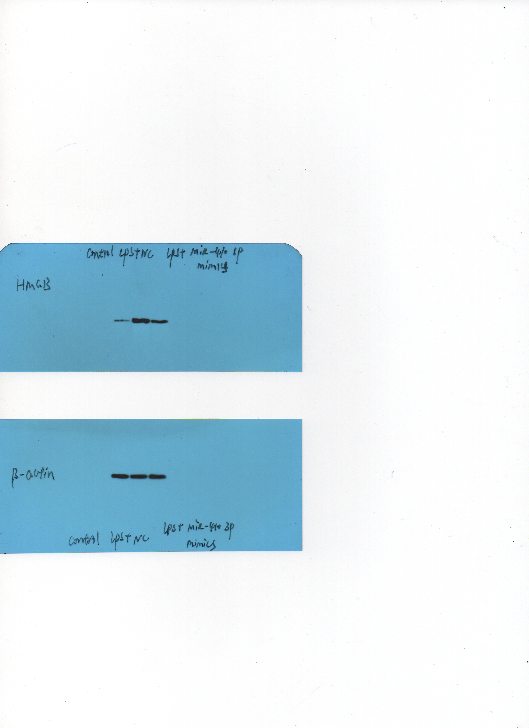
**

**Western blot of Figure 4B: HMGB1 in control, miR-410-3p mimics + empty vector, and miR-410-3p mimics + HMGB1 vector mouse primary chondrocytes.**

**
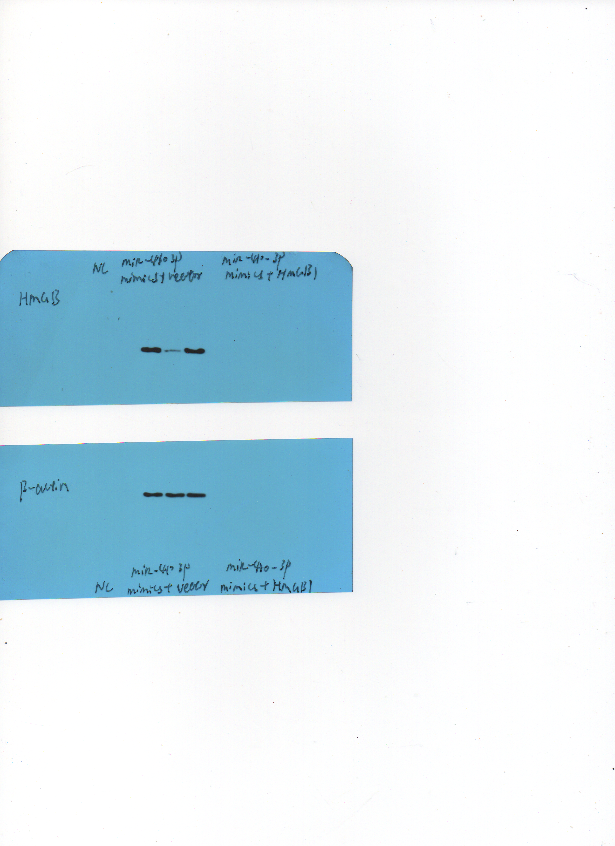
**

**Western blot of Figure 5E: HMGB1, IkBα, and p65 in chondrocytes of Sham-operated, OA + LV-NC and OA + LV-miR-410-3p mimics mice.**

**
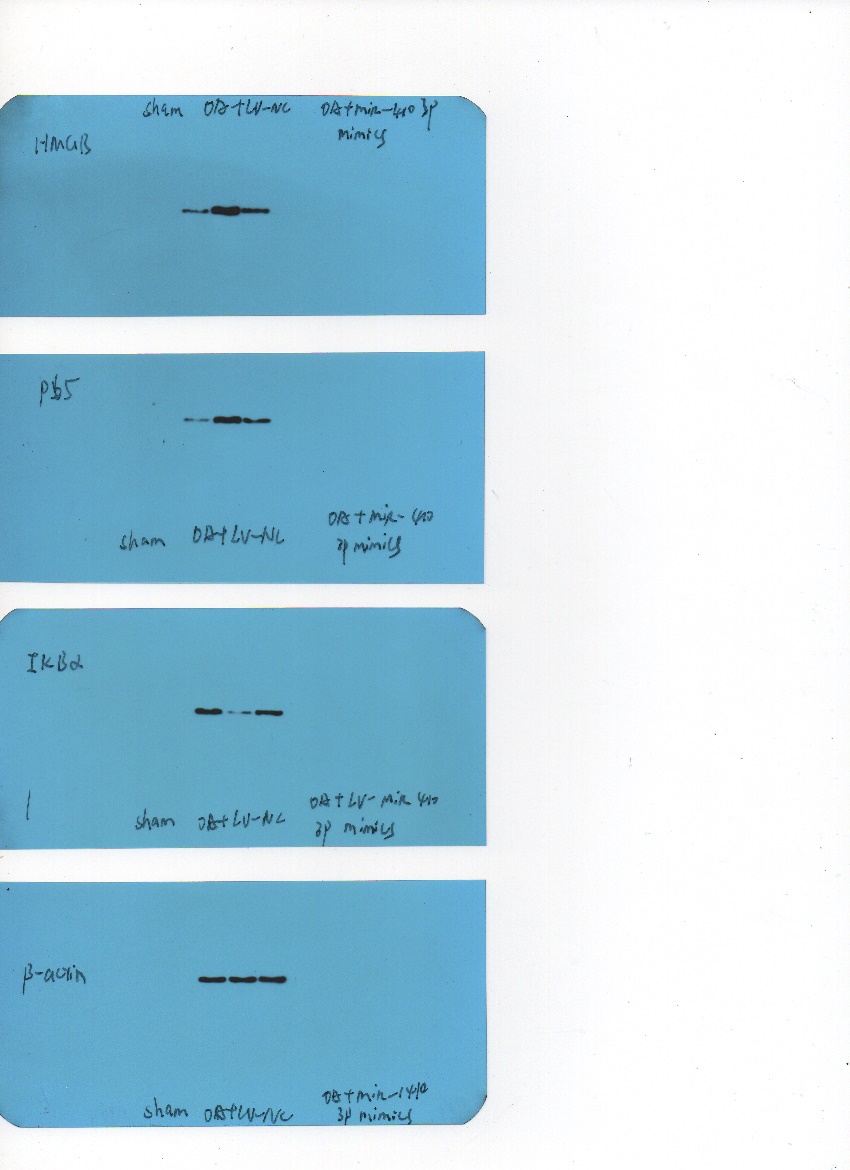
**
